# Mental Health Burden in Different Professions During the Final Stage of the COVID-19 Lockdown in China: Cross-sectional Survey Study

**DOI:** 10.2196/24240

**Published:** 2020-12-02

**Authors:** Junfeng Du, Gwendolyn Mayer, Svenja Hummel, Neele Oetjen, Nadine Gronewold, Ali Zafar, Jobst-Hendrik Schultz

**Affiliations:** 1 Liyuan Hospital Tongji Medical College Huazhong University of Science and Technology Wuhan China; 2 Department of General Internal Medicine and Psychosomatics Heidelberg University Hospital Heidelberg Germany

**Keywords:** mental health, COVID-19, China, depression, anxiety, lockdown, coping strategies, stressors, stress, doctors, nurses, students, media consumption, WeChat

## Abstract

**Background:**

COVID-19 resulted in considerable mental health burden in the Chinese general population and among health care workers at the beginning and peak of the pandemic. However, little is known about potentially vulnerable groups during the final stage of the lockdown.

**Objective:**

The aim of this survey study was to assess the mental health burden of different professions in China in order to find vulnerable groups, possible influencing factors, and successful ways of coping during the last 4 weeks of the lockdown in Hubei Province.

**Methods:**

A cross-sectional online survey asked participants about current residence, daily working hours, exposure to COVID-19 at work, and media preferences. We used a shortened version of the Depression, Anxiety and Stress Scale (DASS-21) to assess mental health. Further assessments included perceived stress (Simplified Chinese version of the 14-item Perceived Stress Scale), coping strategies for all participants, and specific stressors for health care workers. We followed the reporting guidelines of the STROBE (Strengthening the Reporting of Observational Studies in Epidemiology) statement for observational studies.

**Results:**

The sample (N=687) consisted of 158 doctors, 221 nurses, 24 other medical staff, 43 students, 60 teachers/government staff, 135 economy staff, 26 workers/farmers, and 20 professions designated under the “other” category. We found increased depression (n=123, 17.9%), anxiety (n=208, 30.3%), and stress (n=94, 13.7%) in our sample. Professions that were vulnerable to depression were other medical staff and students. Doctors, nurses, and students were vulnerable to anxiety; and other medical staff, students, and economy staff were vulnerable to stress. Coping strategies were reduced to three factors: active, mental, and emotional. Being female and emotional coping were independently associated with depression, anxiety, or stress. Applying active coping strategies showed lower odds for anxiety while mental coping strategies showed lower odds for depression, anxiety, and stress. Age, being inside a lockdown area, exposure to COVID-19 at work, and having a high workload (8-12 hours per day) were not associated with depression, anxiety, or stress. WeChat was the preferred way of staying informed across all groups.

**Conclusions:**

By the end of the lockdown, a considerable part of the Chinese population showed increased levels of depression and anxiety. Students and other medical staff were the most affected, while economy staff were highly stressed. Doctors and nurses need support regarding potential anxiety disorders. Future work should focus on longitudinal results of the pandemic and develop targeted preventive measures.

## Introduction

### Background

In December 2019, pneumonia cases of unknown etiology in Wuhan, Hubei Province, were reported by Chinese authorities. By January 3, 2020, 44 cases requiring hospitalization were officially confirmed [1]. The disease was titled COVID-19 by the World Health Organization in February 2020 [2]. As the infection spread rapidly all over the country [3], the public health response of the government included the largest quarantine in China’s history. Starting from January 23, the lockdown in Hubei Province, the epicenter of COVID-19, lasted 76 days and ended on April 8, 2020 [4]. Zhejiang was another province that was put under lockdown [5].

The pandemic put a considerable psychological burden on citizens, which was not simply due to fear of infection but also isolation, helplessness, and grief over the loss of relatives without having the opportunity to take leave or to organize a funeral. Even more aggravating was that trusted persons, like family and friends, could be infected, and thus, became part of an invisible danger [6].

Previous epidemics, like the severe acute respiratory syndrome (SARS) in Hong Kong in 2003 or the Middle East respiratory syndrome (MERS) in Saudi Arabia in 2012, have taught us to care for the mental health of the general population and frontline health care workers [7-10]. In Hong Kong, suicide rates among older adults increased significantly in 2003 and 2004 [11]. It is noteworthy to recall that high-risk health workers suffered from long-lasting depression and posttraumatic stress responses even 1 year after the SARS outbreak [12].

### Mental Health and Coping Strategies

Several studies have investigated the mental health consequences of the ongoing pandemic in the Chinese population and its strategies to successfully cope with the demanding situation. Wang et al [13] found increased anxiety in nearly 30% of 1210 participants, especially caused by worrying about family members. The same authors also reported longitudinal results, repeating the measures after 4 weeks in March 2020 [14], and found no changes in the scores despite increased infection rates. A high level of trust in doctors and health information, as well as personal protective measures, was rated as helpful. The harmful effects of hoarding food and medicine were described as being associated with elevated levels of depression at the beginning of the crisis [15]. Another study found that cognitive coping behavior (eg, obtaining knowledge about COVID-19) and prosocial coping styles (eg, adherence to social distancing) proved to be protective for the population [16]. These authors described the high impact of the pandemic on the livelihood of the population and examined the harmful effects of media exposure on mental health.

Besides the obvious impact of the pandemic on mental health like the fear of infection and isolation due to quarantine measures [17], excessive media consumption was linked to mood disorders during the lockdown in China [18,19]. However, little is known about the influence of media preferences on the mental health of the population during the crisis.

### The Mental Health of Vulnerable Groups During COVID-19

Since health care workers at the frontline were exposed to particularly demanding conditions during the peak of the pandemic, their mental health and coping strategies have become an early issue of concern. One of the first studies on this topic focused on medical and nursing staff in Wuhan and found elevated levels of subthreshold mental health disturbances in nearly 40% of the 994 participants surveyed [20]. A nationwide study in February reported nearly 5% of medical and nonmedical staff with moderate and high levels of anxiety and about 13% with depression [21]. In this study, nurses and young personnel were found to be particularly at risk for mental distress. A nationwide survey by the end of February showed even higher levels (anxiety: 13%; depression: 12%; insomnia: 38%) for doctors and nurses compared to nonmedical health workers [22]. Risk factors included living in a rural area, being female, and exposure to COVID-19. Additionally, health care workers were burdened by specific clinical and nonclinical stressors (eg, fear of bringing the virus home to family members and the experience of losing colleagues) [23].

There were some specific results on the psychological burden felt by nurses. Nurses in Anhui showed strong emotional responses. Increased exposure to COVID-19 cases evoked more anxiety and anger [24]. Increased levels of insomnia were reported among nurses in Wuhan, which might have been caused by comorbid sleep apnea due to stress [25]. Despite showing symptoms of severe distress, these Wuhan nurses refused to accept psychological help at the beginning of the pandemic [26].

Another vulnerable group included students, the majority of whom lived in quarantine with their families and reported victimization by facing or witnessing various stressful events related to COVID-19 [27]. Other studies found that the COVID-19 crisis impacted sleep quality [28], and increased anxiety among students was reported even after the lockdown ended [29]. Teachers were also affected not only by the outbreak but also by the stress experienced by their pupils [30].

In spite of the many studies regarding the mental health of the general population and health care workers on the frontline of the pandemic, we found no data on further vulnerable groups and professions that may be mentally or emotionally affected by indirect means. Although Huang and Zhao [31] found comparable depressive symptoms among employees in enterprises as in health care workers, and Wang et al [13,14] reported longitudinal results in February and March, these results were from the initial stages of the lockdown and data from the end of the lockdown are missing to date.

### Objectives of the Study

The aim of this survey study was to assess the psychological burden of COVID-19 on the mental health of the Chinese population during the last 4 weeks of the lockdown in Hubei Province. We examined different professions in order to find vulnerable groups, possible influencing factors, and successful ways of coping. Moreover, we looked for specific stressors among doctors and nurses.

## Methods

### Study Design

We used a cross-sectional online survey design in order to investigate the impact of the COVID-19 pandemic on the mental health, stress, specific stressors, and coping strategies of different groups of the Chinese population. The study team of Heidelberg University Hospital developed the concept and the questionnaire, which was translated into Chinese. Its implementation into an online format and sampling was carried out by a publicity enterprise in Wuhan. The Tongji Medical College of the Huazhong University of Science and Technology supported the study by disseminating the link. The study started on March 19 and data were included until April 7. The lockdown in Hubei was officially ended on April 8 by the government [4].

Ethical approval for this study was granted by the Ethics Commission of the Medical Faculty of Heidelberg (S-361/2020). We followed the reporting guidelines of the STROBE (Strengthening the Reporting of Observational Studies in Epidemiology) statement for observational studies [32].

### Measures

The questionnaire was derived from validated instruments and structured into four major parts. The first part asked for demographic data (place of residence, gender, age, marital status, educational background, and occupation), exposure to people infected with COVID-19 in general and at work, working hours per day, and media platforms used to obtain information (multiple choice). The second part asked for mental health parameters like depression, anxiety, and stress, measured by a shortened version of the 21-item Depression, Anxiety and Stress Scale (DASS-21) using a 4-point Likert scale [33]. We used the validated Chinese translation [34]. The instrument refers to a time span of the past week and has been shown to distinguish well between symptoms of depression, anxiety, and stress in clinical and nonclinical samples [35]. In order to assess stress levels during the past month, we used the 14-item Perceived Stress Scale (PSS-14) that explicitly refers to a longer time span using a 5-point Likert scale [36]. Again, we used a validated translation (ie, CPSS-14) [37]. The third part of our questionnaire targeted health care workers only and consisted of a questionnaire that was used in a former study on the SARS outbreak in 2003 [38]. The items asked for specific disease-related stressors of doctors and nurses and were rated on a 4-point Likert scale (0=not at all; 1=slightly; 2=moderately; 3=very much). Finally, the fourth part was again available for all participants and asked for successful coping strategies. The items were again taken from the SARS study [38] and recorded the frequency of use of various coping strategies on a 4-point scale (0=almost never; 1=sometimes; 2=often; 3=almost always). Both scales were translated by a Chinese native speaker (JD).

### Data Analysis

The responses of the participants were downloaded from the online survey tool and further processed and analyzed using SPSS 24 (IBM Corp) [39]. We collected 1006 data sets and removed all data sets that were filled out after April 7 (n=74) and all questionnaires filled out in less than 513 seconds (n=226), which corresponded to the lowest percentile of the mean processing time for all samples. In these cases, we assumed a lack of credibility if a participant took less than this amount of time. Finally, we removed all participants who were younger than the legal age (n=7) and who were not in China during the survey (n=9). We calculated descriptive statistics and reported frequencies, means, standard deviations, and percentages.

Participants answering from Hubei and Zhejiang provinces were regarded as being affected by the lockdown (n=460). All other participants were not directly affected by the lockdown (n=226).

The scoring of the DASS-21 are calculated as sum scores that have to be multiplied by two. The total depression subscale score was divided into normal (0-9), mild (10-13), moderate (14-20), severe (21-27), and extremely severe depression (28+). The anxiety subscale score was divided into normal (0-7), mild (8-9), moderate (10-14), severe (15-19), and extremely severe anxiety (20+). The total stress subscale score was divided into normal (0-14), mild (15-18), moderate (19-25), severe (26-33), and extremely severe stress (34+). Next, we grouped the levels of severity into normal–mild and moderate–extremely severe for each score. We decided to put mild symptoms into one group together with the normal level, since we considered mild symptoms of depression and anxiety to exist regardless of the pandemic [40].

The CPSS-14 scores were calculated by sum scores as well. We reported the CPSS-14 scores and DASS-21 scores nationwide for each profession. For deeper analysis we calculated Pearson correlations in order to assess the relationship of perceived stress during the past 4 weeks and mental health scores for depression, anxiety, and stress during the past week.

The coping strategies and major stressors were calculated as means and standard deviations. We carried out a factor analysis (principal component analysis [PCA] with varimax rotation) for all coping strategies. The Kaiser-Meyer-Olkin (KMO) and Bartlett Test indicated a sufficient cohesion of the variables (KMO=0.76) [41]. Finally, binary logistic regression models were calculated and thereby investigated the associations of gender, lockdown area, contact with COVID-19 infections at work, and coping strategies (factors) with the odds of belonging to moderate–extremely severe depression, anxiety, or stress group. A nonsignificant value of *P*=.40 in the Hosmer-Lemeshow test indicated the goodness of fit of the models [42], and a Nagelkerkes R² of 0.17 showed an acceptable coefficient of determination [41].

In all analyses, *P* values <.05 were considered statistically significant.

## Results

### Participants

The sample included 687 participants, 72.3% (n=496) of whom were female and 27.7% were (n=190) male. The mean age was 36.92 years (SD 9.83) with a range of 18-71 years. The participants consisted of doctors (n=158, 23.0%), nurses (n=221, 32.2%), other medical staff (n=24, 3.5%), students (n=43, 6.6%), teachers/government staff (n=60, 8.7%), economy staff (n=135, 19.7%), workers/farmers (n=26, 3.8%), and others (n=20, 2.9%). We combined doctors and dentists into one category. Other medical staff referred to health care professionals who were not doctors or nurses. Economy staff consisted of employees and self-employed individuals in the IT (information technology) and finance sectors.

A majority of the participants were from Hubei Province (n=449, 65.4%); 30 (4.4%) came from Jiangsu and 21 (3.1%) each from Shanxi and Guangdong. A small group (n=11, 1.6%) came from Zhejiang, which was affected by a lockdown like Hubei. Demographic characteristics and details of each professional group are summarized in Table 1.

**Table 1:** Demographic characteristics of the study participants.

Characteristics		Participants, n (%)
Age (years), mean (SD)		36.92 (9.83)
**Gender**		
	Male	190 (27.7)
	Female	496 (72.3)
**Family status**		
	Single	146 (21.3)
	Married	501 (72.9)
	Divorced	30 (4.4)
	Widowed	2 (0.3)
	In a relationship	8 (1.2)
**Children**		
	Yes	499 (72.6)
	No	188 (27.4)
**Level of education**		
	Middle school	10 (1.5)
	High school	25 (3.6)
	Junior college	168 (24.5)
	Bachelor	384 (55.9)
	Master	77 (11.2)
	Doctorate	23 (3.3)
**Profession**		
	Doctors/dentists	158 (23.0)
	Nurses	221 (32.2)
	Other medical staff (eg, volunteers, pharmacists, midwives)	24 (3.5)
	Students	43 (6.3)
	Teachers/government staff	60 (8.7)
	Economy (eg, employees, self-employed, salespersons)	135 (19.7)
	Workers/farmers	26 (3.8)
	Others (eg, housewives)	20 (2.9)
**Residence**		
	Hubei	449 (65.4)
	Jiangsu	30 (4.4)
	Guangdong	21 (3.1)
	Shanxi	21 (3.1)
	Shandong	17 (2.5)
	Fujian	16 (2.3)
	Sichuan	15 (2.2)
	Shanghai	15 (2.2)
	Hunan	14 (2.0)
	Zhejiang	11 (1.6)
	Provinces with less than 10 participants	78 (11.2)
Total		687 (100)

### Perceived Stress and Mental Health

Perceived stress was measured with a mean score of 23.70 (SD 7.52). The mean values for DASS-21 depression was 6.62 (SD 7.80), for DASS-21 anxiety was 7.01 (SD 7.00), and for DASS-21 stress was 10.18 (SD 8.63). Perceived stress was significantly correlated with DASS-21 depression (*r*=0.61, *P*<.001), DASS-21 anxiety (*r*=0.57, *P*<.001), and DASS-21 stress (*r*=0.66, *P*<.001).

Findings on mental health status for each profession are reported in Table 2; the DASS-21 scores were put into categories normal–mild and moderate–extremely severe.

**Table 2:** Results of the Simplified Chinese version of the 14-item Perceived Stress Scale (CPSS-14) and the Depression, Anxiety and Stress Scale - 21 Items (DASS-21).

Profession	Participants, n	CPSS-14		DASS-21 depression			DASS-21 anxiety		DASS-21 stress	
		Mean (SD)	NM^a^, n (%)	MES^b^, n (%)	NM, n (%)	MES, n (%)		NM, n (%)		MES, n (%)
Doctors	158	23.16 (7.26)	134 (84.8)	24 (15.2)	106 (67.1)	52 (32.9)		138 (87.3)		20 (12.5)
Nurses	221	23.62 (7.19)	183 (82.8)	38 (17.2)	152 (68.8)	69 (31.2)		197 (89.1)		24 (10.9)
Other medical staff	24	22.25 (8.09)	19 (79.2)	5 (20.8)	17 (70.8)	7 (29.2)		20 (83.3)		4 (16.7)
Students	43	26.30 (7.79)	33 (76.7)	10 (23.3)	25 (58.1)	18 (41.9)		34 (79.1)		9 (20.9)
Teachers/ govt staff	60	22.98 (6.09)	51 (85.0)	9 (15.0)	44 (73.3)	16 (26.7)		53 (88.3)		7 (11.7)
Economy staff	135	23.93 (8.68)	108 (79.4)	27 (20.0)	102 (75.6)	33 (24.4)		112 (83.0)		23 (17.0)
Workers/farmers	26	23.15 (6.69)	21 (77.8)	5 (19.2)	19 (73.1)	7 (26.9)		23 (85.5)		3 (11.5)
Others	20	26.25 (7.62)	15 (75.0)	5 (25.0)	14 (70.0)	6 (30.0)		16 (80.0)		4 (20.0)
Total	687	23.70 (7.52)	564 (82.1)	123 (17.9)	479 (69.7)	208 (30.3)		593 (86.3)		94 (13.7)

^a^NM: normal–mild.

^b^MES: moderate–extremely severe.

### Working Hours Per Day

The majority of the participants reported working 4-8 hours per day (n=427, 61.4%). This was the case in the following groups—nurses: 145/221, 65.6%; students: 39/43, 90.7%; teachers/government staff: 47/60, 78.4%; economy staff: 97/135, 71.9%; workers/farmers: 18/26, 69.3%; others: 15/20, 75.0%.

A sizeable part of the sample reported working 8-12 hours per day (n=260, 37.4%). This high workload typically affected doctors (103/158, 65.2%) and other medical staff (13/24, 54.2%).

### Contact With COVID-19 at Work

In total, 6 (0.9%) participants were infected themselves (2 doctors, 3 nurses, and 1 member of the group teachers/government staff). Of all participants, 180 (26.2%) had contact with people infected by the virus at work. The most affected group were doctors (68/158, 43.0% had contact with COVID-19 at work), followed by other medical staff (10/24, 41.7%), nurses (88/221, 39.8%), teachers/government staff (8/60, 13.3%), economy staff (5/135, 3.8%), workers/farmers (1/26, 3.8%). Participants from the other professions category and students did not report contact with COVID-19 at work.

### Media Preferences

When asked about the primary way participants obtained information in the past month, the majority of respondents indicated having done so through WeChat (n=606, 88.2%) (Table 3).

**Table 3:** Participants’ answers to the multiple-choice question: what was your main way of obtaining information during the last month?

Source	Participants, n (%)
Newspaper	53 (7.71)
Television	465 (67.69)
Weibo	304 (44.25)
WeChat	606 (88.21)
Circle of friends^a^	502 (73.07)
Family/colleagues	311 (45.27)
Other	104 (15.14)

^a^Includes WeChat groups and other social media–related groups.

### Coping Strategies

The three most successful ways of facing the demands of COVID-19 in daily life and work, out of 12 possible answers, were taking protective measures (mean 2.57, SD 0.67), actively acquiring more knowledge about COVID-19 (mean 2.09, SD 0.78), and engaging in recreational activities (mean 1.94, SD 0.77*).* All coping strategies are listed in Table 4.

Three dimensions could be extracted after carrying out the PCA and were named as active coping, mental coping, and emotional coping, after analyzing the content of the items. The dimensions accounted for 47.2% of the variance (Table 4).

**Table 4:** Matrix of coping strategy components and three statistics after varimax rotation (the rotation is converged in five iterations; method of extraction: main component analysis).

Items		Mean (SD)	Factor loadings		
			Active	Mental	Emotional
**Coping strategy components**					
	Taking protective measures (washing hands, wearing a mask, taking one’s own temperature, etc)	2.57 (0.67)	0.77	–0.03	0.01
	Actively acquiring more knowledge about COVID-19 (symptoms, transmission pathway, etc)	2.09 (0.78)	0.75	0.17	0.02
	Changing one’s thoughts and facing the situation with a positive attitude	1.90 (0.83)	0.53	0.44	–0.12
	Engaging in recreational activities (WeChat, Weibo, TikTok, online shopping, online movies, exercises)	1.94 (0.77)	0.42	0.23	–0.06
	Video chatting with family and friends by phone to share concerns and support	1.69 (0.80)	0.40	0.43	–0.02
	Engaging in health-promoting behaviors (more rest, exercise, balanced diet, etc)	1.76 (0.82)	0.27	0.68	–0.06
	Acquiring mental health knowledge and information	1.36 (0.91)	0.27	0.63	0.01
	Practicing relaxation methods (meditation, yoga, Tai Chi, etc)	0.88 (0.85)	–0.07	0.83	0.09
	Limiting oneself from watching too much news about COVID-19	0.53 (0.73)	–0.11	0.13	0.72
	Distracting oneself from thinking about COVID-19 issues by suppression or keeping busy	0.70 (0.82)	0.03	0.10	0.75
	Venting emotions by crying, screaming, smashing things, and so on	0.23 (0.48)	–0.08	–0.08	0.53
	Using alcohol or drugs	0.22 (0.53)	0.07	–0.10	0.53
**Statistics**					
	Eigenvalue	—^a^	2.89	1.71	1.07
	Percentage of total variance	—	24.05	14.23	8.93
	Total variance	—	—	—	47.21

^a^Not applicable.

### Predictors of Increased Levels of Depression, Anxiety, and Stress

We calculated three binary logistic regression models in order to find associations of gender, lockdown area, contact with COVID-19 infection at work, and coping factors with the odds of belonging to the group for moderate–extremely severe depression, anxiety, or stress. Being female and applying emotional coping strategies increased the probability of belonging to the moderate–extremely severe depression, anxiety, or stress group. Applying active coping strategies reduced the probability of being affected by moderate–extremely severe anxiety, while mental coping strategies reduced the probability in all three moderate–extremely severe mental health groups. Age, being in a lockdown area, having contact with COVID-19 at work, and having a high workload (8-12 hr per day) did not significantly predict the odds of expressing moderate–extremely severe symptoms of depression, anxiety, or stress. The results are displayed in detail in Table 5.

**Table 5:** Results of a logistic regression predicting the probability of experiencing moderate–extremely severe (MES) depression, anxiety, or stress.

Variable	MES depression		MES anxiety		MES stress	
	B (SE)	OR^a^ (95% CI)	B (SE)	OR (95% CI)	B (SE)	OR (95% CI)
Gender (female)	0.81 (0.27)	2.24 (1.33- 3.77)	0.47 (0.22)	1.61 (1.05-2.47)	0.78 (0.30)	2.19 (1.21-3.96)
Age	0.01 (0.01)	1.01 (0.98-1.03)	–0.01 (0.01)	0.99 (0.97-1.00)	–0.01 (0.01)	0.99 (0.96-1.01)
Hubei/Zhejiang	–0.43 (0.25)	0.65 (0.40-1.05)	–0.07 (0.21)	0.93 (0.61-1.41)	–0.22 (0.28)	0.80 (0.46-1.39)
Contact with COVID-19 infection at work	0.07 (0.26)	1.08 (0.64-1.80)	0.15 (0.49)	1.16 (0.76-1.79)	–0.03 (0.30)	0.97 (0.54-1.74)
Daily workload (8-12 hr)	0.06 (0.22)	1.06 (0.69-1.65)	0.23 (0.19)	1.26 (0.87-1.83)	0.32 (0.25)	1.38 (0.85-2.25)
Active coping	–0.14 (0.11)	0.87 (0.71-1.07)	–0.21 (0.09)	0.81 (0.68-0.97)	–0.11 (0.12)	0.89 (0.70-1.13)
Mental coping	–0.56 (0.12)	0.57 (0.45-0.72)	–0.42 (0.10)	0.67 (0.55-0.81)	–0.67 (0.14)	0.51 (0.39-0.67)
Emotional coping	0.63 (0.10)	1.89 (1.55-2.30)	0.80 (0.10)	2.16 (1.80-2.60)	0.82 (0.11)	2.27 (1.81-2.84)

^a^OR: odds ratio.

### Major Stressors (Health Care Workers Only)

Out of 18 stressors, the three most demanding aspects for health care workers (n=375) were related to worries about infecting one’s family with COVID-19 (mean 1.46, SD 0.86), followed by the potential deterioration of their patients’ condition (mean 1.42, SD 0.79) and their patients’ emotional reaction (mean 1.3, SD 0.81) (Table 6).

**Table 6:** Doctors’ and nurses’ responses to the question: when you think about COVID-19 in your life and work, how often did you think or worry about the following things? (0=not at all, 3=very much) (n=375).

Stressor	Response, mean (SD)
Worries about infecting your family with COVID-19	1.46 (0.86)
Deterioration of patients’ condition	1.42 (0.79)
Patients’ emotional reaction	1.30 (0.81)
Emotional reaction of patients’ families	1.29 (0.79)
Uncertainties about when the epidemic will be under control	1.27 (0.78)
Coworkers displaying COVID-19–like symptoms	1.27 (0.79)
Worries about getting infected	1.24 (0.78)
Worries about being negligent and endangering patients	1.23 (0.88)
Worries about lack of proper knowledge and equipment	1.23 (0.79)
Worries about being negligent and endangering coworkers	1.18 (0.83)
Worries about nosocomial spread	1.15 (0.82)
Conflict between duty and safety	1.15 (0.81)
Being infected by colleagues	1.12 (0.81)
Protective gears being a hinderance to providing quality care	1.12 (0.80)
Being blamed by supervisors/managers	1.10 (0.80)
Displaying COVID-19–like symptoms yourself	1.09 (0.77)
Worries about the lack of manpower	1.07 (0.91)
Being without a properly equipped environment	1.05 (0.84)
Physical discomfort caused by protective gears	1.01 (0.79)
Ambiguity in the responsibilities between doctors and nurses	1.00 (0.86)
Frequent modification of infection control procedures	0.96 (0.81)
Coworkers being emotionally unstable	0.96 (0.77)
Unclear documentation and reporting procedures	0.92 (0.78)

## Discussion

### Predictors of Mental Health Symptoms

This survey aimed to assess the psychological burden and mental health of the Chinese population during the final stages of the lockdown, as well as to determine successful coping strategies and potentially vulnerable professional groups with specific support needs. Our results suggest that being female and, independent of gender, applying certain coping strategies increased the incidence of symptoms of depression, anxiety, and stress. Emotional coping strategies like venting emotions, consuming alcohol, or limiting oneself from information were not helpful for participants dealing with COVID-19–related psychological problems. Active strategies to cope with moderate–extremely severe anxiety, such as taking protective measures and acquiring more knowledge were more beneficial, but the most effective strategy was focusing on mental coping like relaxation techniques and gaining knowledge about mental health. Our results confirm the findings of Guo et al [16], who used another instrument to determine coping strategies but found emotion-focused strategies to worsen mental health, while cognitive and problem-focused coping strategies to be helpful. Interestingly, exposure to COVID-19 at work, living within a lockdown area, and daily workload did not play a significant role in predicting elevated symptoms.

We found no overall increased mean values in perceived stress and depression, anxiety, and stress in comparison to former (pre–COVID-19) samples (eg, compared to the perceived stress levels of patients in Hong Kong [43] or among residents in Beijing [44]) regarding mental health scores. This could be because the Chinese population had already become accustomed to the burden of COVID-19 by the end of the lockdown. The first studies in January investigating the psychological impact of the outbreak on the population reported high levels of burden [16,31,45]. Wang et al [13,14] measured DASS-21 scores twice at the beginning and at the peak of the pandemic in China and found increased anxiety in nearly 30% of participants; yet no longitudinal increases were noted. In our study, we added a third point of measurement at the final stage of the lockdown and found a considerable share of people who expressed moderate to extremely severe symptoms of depression (17.9%), anxiety (30.3%), and stress (13.7%). This result should be taken seriously as our sample revealed a higher percentage of people with increased levels of depression while stress scores were lower compared to the Wang et al [13,14] studies. We also observed a high correlation of perceived stress in the past 4 weeks and actual scores in depression. Previous research on the etiology of depression could show that stress might be one predictor for this mood disorder [46,47]. Following this line of argument, an increase of depressive symptoms by the end of the lockdown could be interpreted as part of a concerning development.

### Vulnerable Groups

Some groups in our sample were more affected by symptoms of depression, anxiety, or stress than others. Students were vulnerable to moderate–extremely severe symptoms in all three categories. Outside the pandemic context, Chinese students have been reported to be affected by mental health problems due to stressful academic demands [48,49]. The consequences of the pandemic on students’ lives were aggravated by infection control measures, online learning on a tight schedule, and uncertain future prospects [50]. A cross-sectional study among medical students after the lockdown revealed high levels of anxiety (38% of participants) and showed that social media played an important role in adherence to protective measures among them [29]. According to our results, the main method of getting COVID-19–related information was WeChat, directly followed by circle of friends, which in China refers to WeChat groups and other social media–related groups.

Economy staff were highly burdened by stress but did not exhibit more depression or anxiety than other groups. This result does not support that of Huang and Zhao [31], who, using different protocols, questioned enterprise employees in early February, and found depression among 20% of participants and anxiety among 34%. Increased stress levels in our sample could be explained by the fact that we put together all employees and self-employed people in the finance and IT sectors as well as salespersons in one group. Enhanced stress may be a result of the concerns to ensure livelihood for their own family during the lockdown, as shown previously [16]. Further differentiated results are needed to allow for a more specific statement.

Doctors and nurses in our sample were highly affected by anxiety; doctors had the highest workload per day. Nevertheless, perceived stress and DASS-21 stress levels were not higher than other groups, which may be due to a high professional devotion, as reported in previous research [51]. Issues about patient care like the deterioration of the patients’ condition and the emotional reaction of patients and their families were perceived as stressful. In another study, nurses in Wuhan were found lacking in training for dealing with uncooperative patients [26]. Several studies have reported on the need for training Chinese medical staff on doctor-patient communication outside the context of the COVID-19 crisis [52]. The anxiety felt by doctors and nurses in our sample may be the result of the fear of bringing COVID-19 to their own families, the most intense stressor in our and other samples [23,51]. A further stressor found in other studies included spending too much time on social media while searching for COVID-19 updates. Doing so promoted symptoms of depression and anxiety in Chinese adults in general as well as in health professionals [19].

Finally, other medical staff, a small group consisting of volunteers, midwives, and pharmacists in our sample, was more affected by depression and stress than other groups and was vulnerable to anxiety as well. During the pandemic, many volunteers supported hospitals in a frontline capacity [53], and our results show that these individuals, who had the second highest workload per day after doctors, have been overlooked so far and should receive more attention since they seem to have special support needs. This group might lack the institutional psychological support that is delivered to the core personnel in a frontline hospital.

### Future Implications

By 2017, there were only 33,400 licensed psychiatrists in China [54]. In recent years, the Chinese National Health Commission has established several mental health initiatives to extend mental health care to the general population. During the COVID-19 pandemic, the commission published guidelines and treatment instructions, uploaded videos of mental health education via WeChat, and established expert emergency groups for mental health services at the hospitals [55].

However, health care workers continue to be in dire need of greater access to specific mental health services [56], and further research is needed on the role of media consumption and mental health during such crises. Although the internet provided many supporting measures like telemedicine for patients with COVID-19 [57] and online mental health education and counseling [58], excessive exposure to the media seems to play a significant role in explaining mental health problems during the pandemic [18]. The Chinese population might find itself in a paradox: acquiring knowledge about the virus and mental health measures is helpful but in order to maximize coverage this knowledge is spread by governmental and other institutions via social media [55,58], which has its own harmful mental consequences [16,19]. As a result, it is not possible for individuals to avoid media consumption. More conclusive findings on the role of the media and the mental health of subgroups of the Chinese population are needed.

### Limitations

Although we received 687 responses, the professional groups in this study were not of equal size; numbers were especially limited for the students and other medical staff categories, which reduces the power of statistical analysis. Targeted investigations may be needed to assess the status of underrepresented professions in a differentiated way. Further, online studies are unable to allow a valuable diagnostic assessment, and this limitation applies to all former studies. This is further aggravated due to the great variability in instruments used in different surveys, which reduces the comparability of results. Some authors used the DASS-21 previously [13,14] but with different cut off-scores (see below), while some relied on the Self-rating Depression Scale (SDS) [45], the Patient Health Questionnaire (PHQ) [56], the Generalized Anxiety Disorder 7-item (GAD-7), the Center for Epidemiology Scale for Depression (CES-D) [31], or the Post-Traumatic Stress Disorders (PTSD) Checklist [16]. However, only a face-to-face diagnostic interview by a qualified medical doctor or a clinical psychologist could allow a statement regarding a mental disorder. Therefore, our results hint at a certain development but should not be interpreted as a diagnosis of the population. Moreover, we compared our results to the studies of Wang et al [13,14], who used slightly different cut-off scores in interpreting the borders between mild and moderate depression and anxiety as suggested originally by Lovibond and Lovibond [33]. However, even without knowing their exact frequencies for single scores, our findings on increases in depression would have been even higher if Wang et al had used the original cut-off scores. Finally, we only asked for media preferences and not for the time spent on media platforms. A detailed analysis on the reasons for media consumption (entertainment, information, relaxation), the way of usage (alone, together), and mental health is necessary to reveal a more holistic picture.

### Conclusion

A considerable part of the general population in China reported elevated symptoms of depression, anxiety, and stress during the final stages of the COVID-19 lockdown. Doctors, nurses, students, and other medical staff were found to be in imminent danger of developing mental health problems. Similarly, economy staff was also highly stressed. Being female was an additional risk factor for potential vulnerability toward developing mental health problems. We recommend providing additional specific information to these subgroups targeting their respective mental health profile and to personalize the successful coping strategies found in our results (ie, active and mental coping). These refer to constructive ways of behavior (eg, actively acquiring knowledge, applying protective measures) and mental health strategies (eg, relaxation techniques, psycho-education, and promoting social contact). Profession-specific mental health prevention programs should be developed and provided in formats preferred by the respective age, gender, or professional groups.

## Abbreviations

CES-D: Center for Epidemiology Scale for Depression
CPSS-14: Simplified Chinese version of the 14-item Perceived Stress Scale
DASS-21: Depression, Anxiety and Stress Scale - 21 Items
GAD-7: Generalized Anxiety Disorder 7-item
KMO: Kaiser-Meyer-Olkin
MERS: Middle East respiratory syndrome
PCA: principal component analysis
PHQ: Patient Health Questionnaire
PSS-14: 14-item Perceived Stress Scale
PTSD Checklist: Post-Traumatic Stress Disorders Checklist
SARS: severe acute respiratory syndrome
SDS: Self-rating Depression Scale
STROBE: Strengthening the Reporting of Observational Studies in Epidemiology
